# Supplementary material for: Comprehensive Integrated Analysis Reveals the Spatiotemporal Microevolution of Cancer Cells in Patients with Bone-Metastatic Prostate Cancer
Source: Biomedicines. 2025 Apr 9;13(4):909. doi: 10.3390/biomedicines13040909 (PMC12024866; doi:10.3390/biomedicines13040909)
Supplement: Supplementary file 1 [file biomedicines-13-00909-s001.zip › biomedicines-3542062-supplementary.pdf]

Supplementary Files

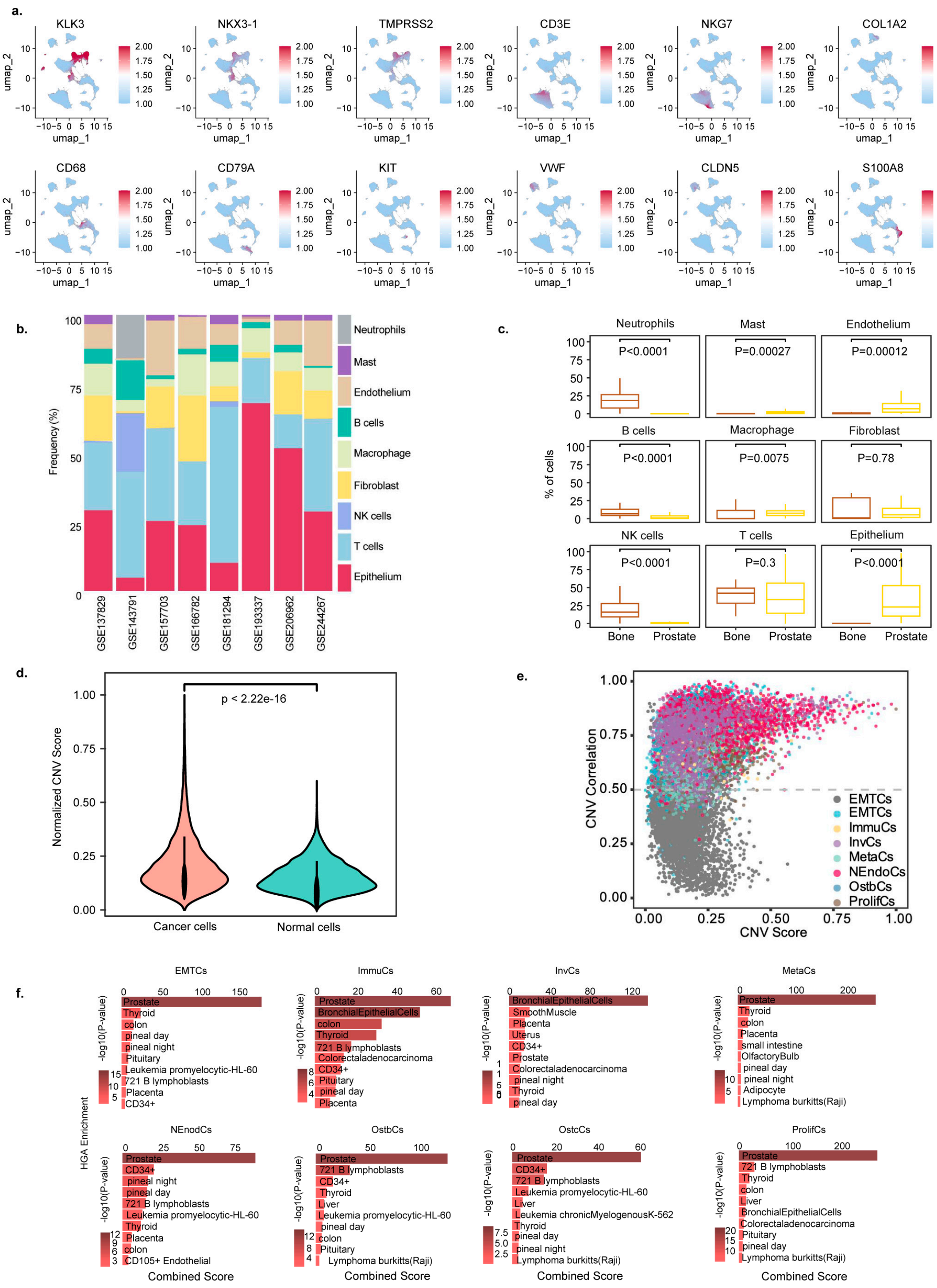

Figure S1. Single cell transcriptome analysis of prostate cancer revealed cell heterogeneity and gene copy number variation (CNV) characteristics. (a) UMAP visualization of key gene expression at the single-cell level, demonstrating distinct expression patterns of marker genes across various cell populations within prostate cancer and its tumor microenvironment. **(b)** Cell type composition across multiple datasets from the GEO database, with stacked bar plots

indicating the relative proportions of different cell types (color-coded). **(c)** Comparative analysis of the proportions of immune cells, fibroblasts, and epithelial cells between primary prostate tumors ("Prostate") and bone metastatic lesions ("Bone"). Statistical significance is indicated by P-values (box-and-whisker plots). **(d)** Violin plot comparing CNV (copy number variation) scores between cancer and normal cells. Cancer cells exhibit significantly higher CNV scores ($p < 2.22e-16$). **(e)** Scatter plot showing the relationship between CNV score and CNV deviation. Points are colored according to cell subtype, illustrating heterogeneity in genomic instability. **(f)** Functional enrichment analysis of different cell subpopulations. Bar plots display the top enriched biological pathways for each cell type based on combined scores.

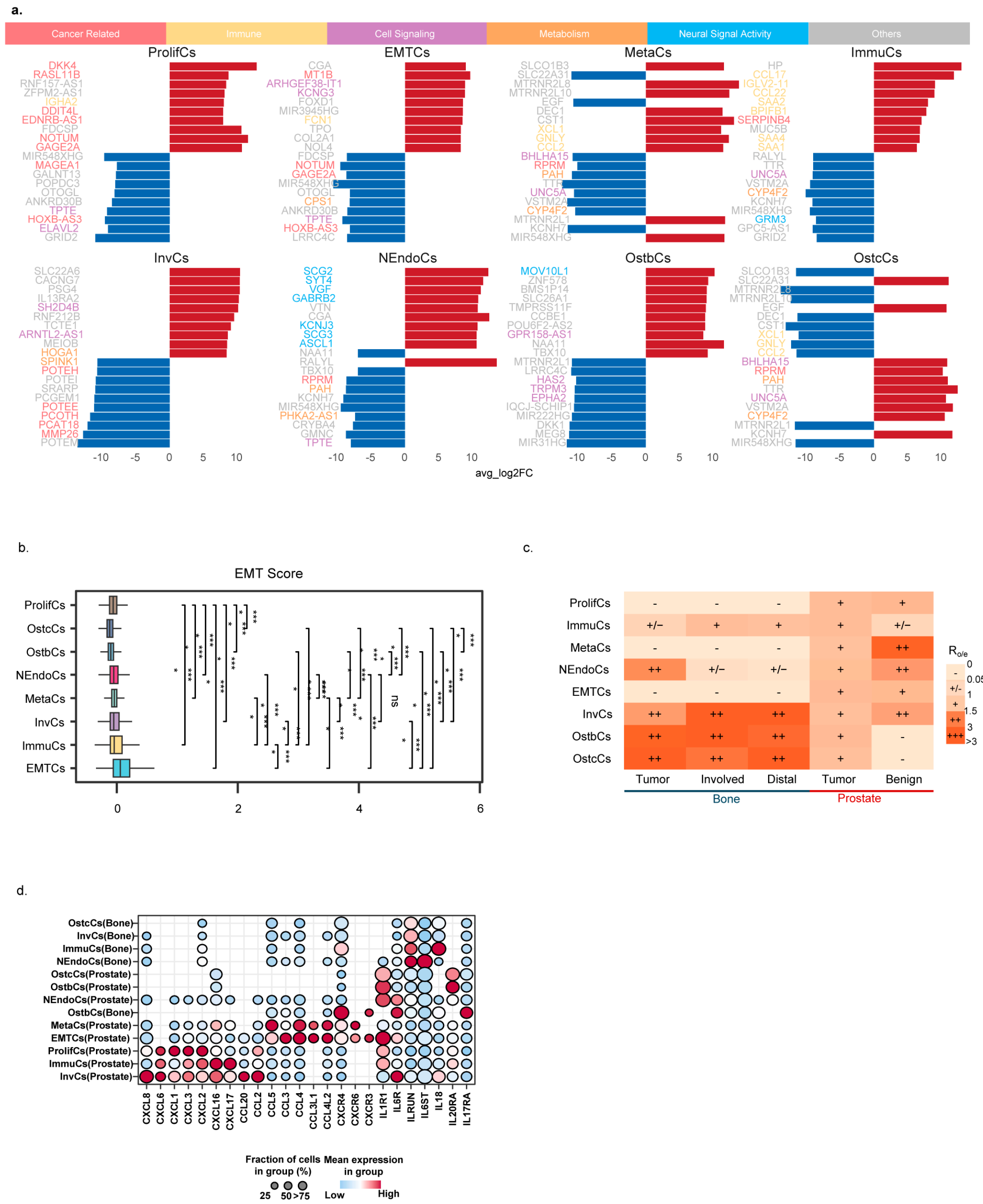

Figure S2. Chemokine expression and EMT characterization of cell subsets in prostate cancer microenvironment. (a) Bar plots illustrating the differential expression of chemokines across various cancer cell subtypes. Red bars indicate upregulated genes and blue bars indicate downregulated ones. The analysis highlights distinct chemokine expression profiles in ProlifCs, EMTCs, MetaCs, ImmuCs, and other functional subsets, reflecting their divergent roles in the tumor microenvironment. **(b)** Boxplots showing the distribution of EMT scores across different

cancer cell subtypes. EMTCs exhibit the highest EMT scores, indicating a strong mesenchymal phenotype, while other subtypes show varied degrees of epithelial or hybrid states. Statistical significance is denoted by asterisks. **(c)** Heatmap summarizing the differential expression and pathway activity of cell subtypes in bone metastasis versus primary prostate tumor tissues. The intensity of color indicates the level of enrichment, with symbols denoting changes: "++" for significantly upregulated, "+" for moderately upregulated, "+/−" for no significant change, and "−" for significantly downregulated. **(d)** Bubble plot presenting the expression of selected genes across different cell subpopulations and tissue origins. Bubble size represents the proportion of expressing cells, while color intensity reflects the mean expression level.

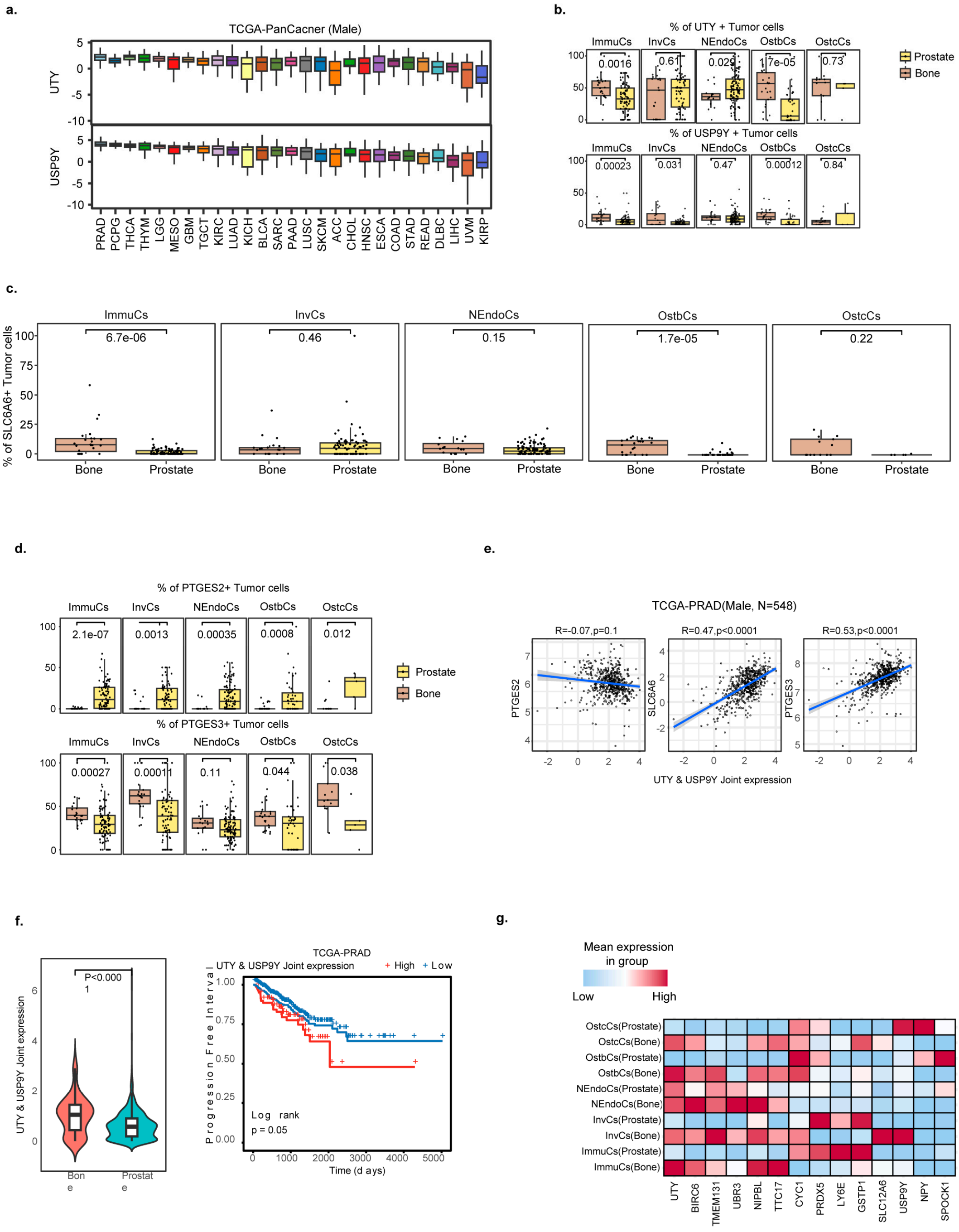

Figure S3. Expression and Prognostic Significance of UTY and USP9Y in Prostate Cancer. (a) Comparative expression analysis of UTY and USP9Y across various cancer types in the TCGA Pan-Cancer dataset, limited to male patients. **(b)** Boxplots depicting the relative proportions of UTY⁺ and USP9Y⁺ tumor cells among different epithelial subtypes in prostate and bone metastatic samples. **(c)** Proportion of SLC6A6⁺ tumor cells within each epithelial subtype, comparing prostate and bone samples. **(d)** Proportions of PGE2+ and PTGES3+ tumor cells in

epithelial subtypes from prostate and bone samples. **(e)** Correlation between UTY and USP9Y co-expression and PTGES3+ tumor cells in TCGA-PRAD. **(f)** Co-expression levels of UTY and USP9Y in prostate and bone samples (violin plots) and survival analysis by co-expression levels (Kaplan-Meier). **(g)** Heatmap depicting the average expression levels of key genes (e.g., UTY, USP9Y, PTGES3, SLC6A6) among various cancer cell subtypes in prostate and bone samples. Red indicates higher expression, while blue indicates lower expression.

a

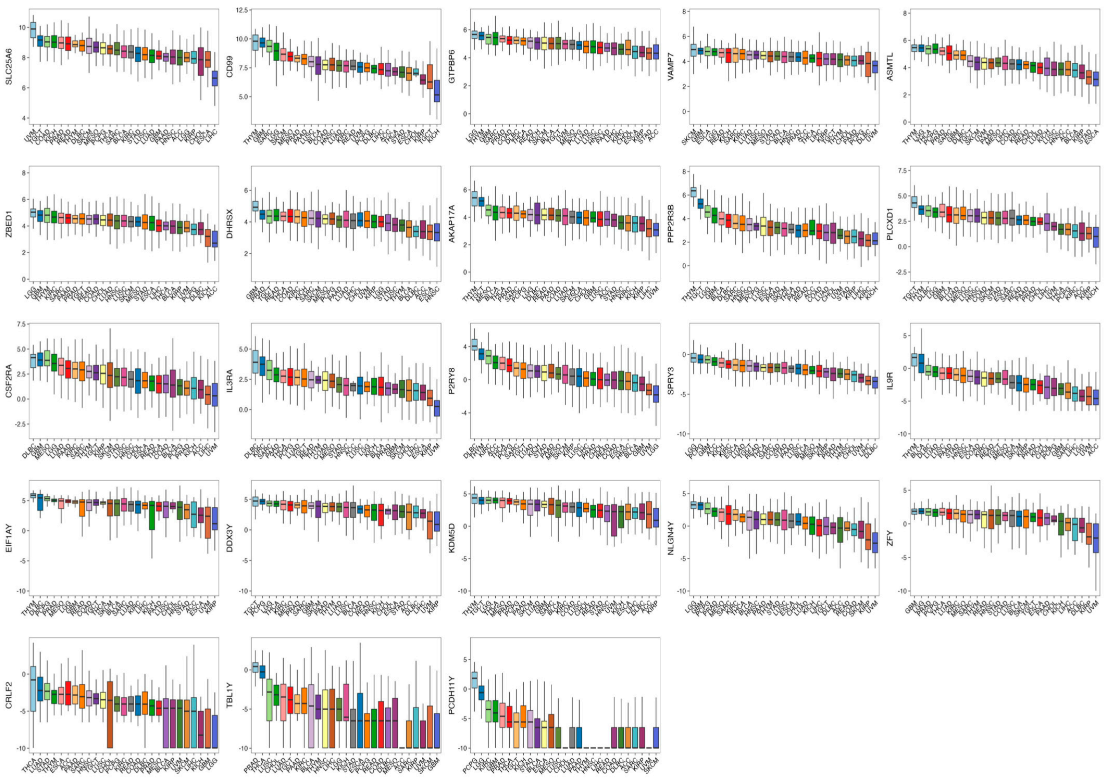

b

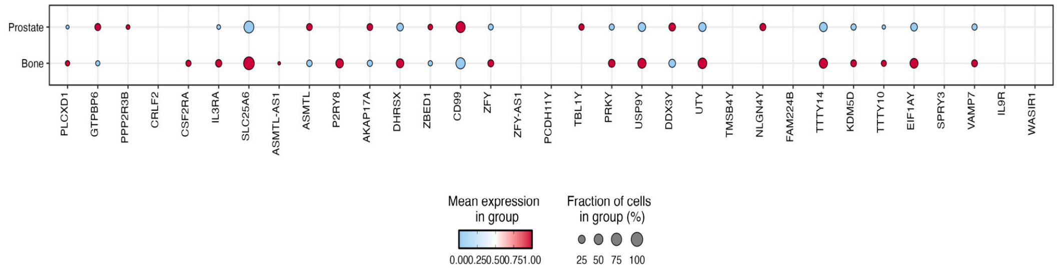

Figure S4. Distinct Gene Expression Profiles Across Cell Subtypes and Metastatic Sites in Prostate Cancer. (a) Box plots show the expression levels of Y chromosome genes in different tumors, revealing significant transcriptional heterogeneity among pancarcinomas. **(b)** Dot plots compare the average amount and proportion of cells expressing specific genes in prostate and bone metastases, highlighting site-specific gene expression differences associated with tumor progression.

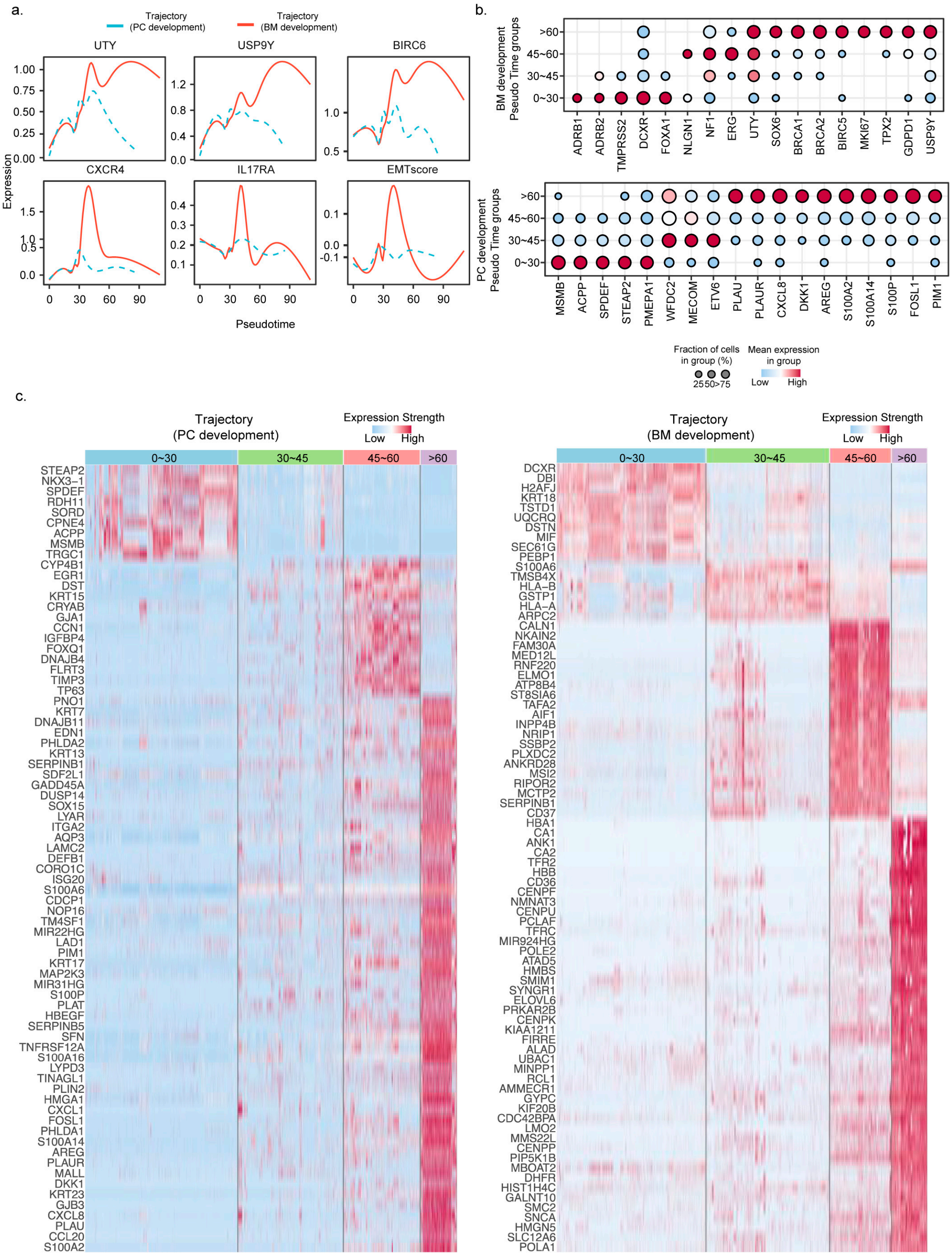

Figure S5. Gene Dynamics in Prostate Cancer Primary Focus and bone Metastasis Progression Trajectory. (a) Changes in the expression of key genes and EMT scores in the progression of prostate cancer primary lesion (PC, blue) and bone metastasis (BM, red) (pseudo-temporal analysis). The vertical axis represents gene expression or EMT score, and the horizontal axis represents pseudo-time progression. **(b)** The proportion of key genes expressed in the development trajectory of prostate cancer (PC) and bone metastasis (BM) (bubble map), the size of the bubble indicates the proportion of expressing cells, and the depth of color indicates the level of gene expression (high expression in red, low expression in blue). **(c)** Gene expression changes in the development trajectory of PC and BM (heat map), horizontal axis represents the pseudo-time process, vertical axis represents the gene name, and color depth represents the gene expression level (red high expression, blue low expression).

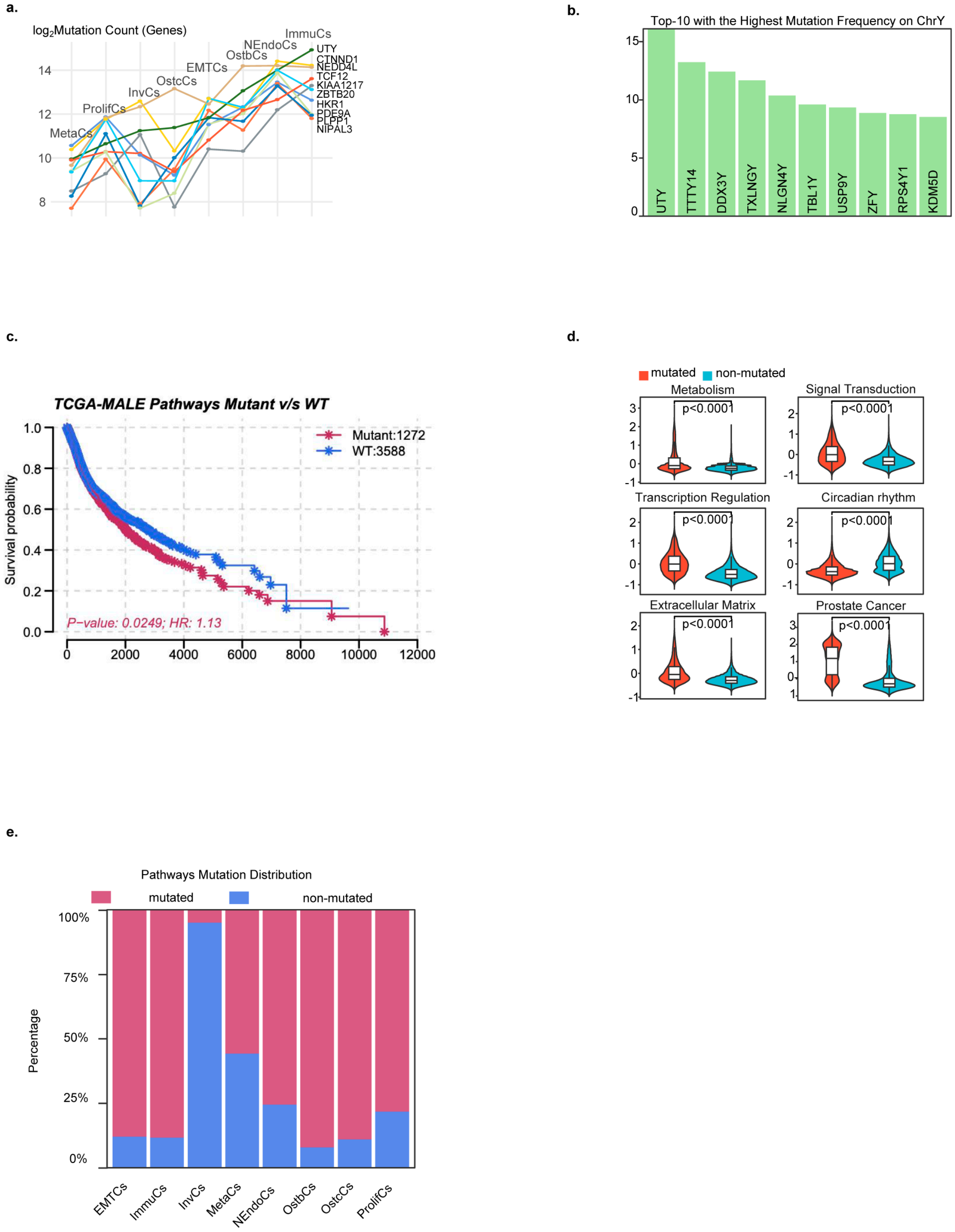

Figure S6. Mutation Landscape and Pathway Alterations in Prostate Cancer Development. (a) Line plot displaying log-transformed mutation counts of selected genes across major prostate cancer cell subtypes. Different lines represent different subtypes, highlighting variability in mutation burden, with higher mutation loads in NEndoCs, OstbCs, and ImmuCs. **(b)** Bar chart showing the top 10 most frequently mutated genes located on the Y chromosome, indicating recurrent male-specific mutational hotspots such as UTY, USP9Y, and TMSB4Y. **(c)** Kaplan–

Meier survival analysis comparing patients with pathway mutations versus wild-type (non-mutated) pathways in the TCGA-PRAD cohort. Patients with mutations showed significantly worse survival ($p = 0.0249$; HR = 1.13). **(d)** Violin plots comparing pathway activity scores between mutated and non-mutated samples. Pathways assessed include metabolism, signal transduction, transcription regulation, circadian rhythm, extracellular matrix, and prostate cancer-specific signaling. Mutated samples consistently show elevated pathway activity ($p < 0.0001$ for all comparisons). **(e)** Stacked bar plots showing the proportion of pathway-mutated and non-mutated cells across different cancer cell subtypes. Certain cell states exhibit a higher burden of pathway mutations, suggesting their involvement in advanced disease stages or therapy resistance.

Table S1 A glossary of terms

Term	Description
**scRNA-seq**	Single-cell RNA sequencing; profiles gene expression at single-cell resolution.
**Cell Ranger**	Pipeline from 10x Genomics for raw data processing, alignment, and gene counting in scRNA-seq.
**Seurat**	R package for scRNA-seq data analysis, including normalization, clustering, and visualization.
**UMAP**	Uniform Manifold Approximation and Projection; used for dimensionality reduction and visualization.
**PCA**	Principal Component Analysis; reduces data dimensionality by capturing variance.
**QC**	Filters out low-quality cells based on metrics such as gene count or mitochondrial content.
**CNV**	Copy Number Variation; changes in the number of copies of genomic DNA segments.
**inferCNV**	R package used to infer CNVs from scRNA-seq data using reference cell populations.
**EMT**	Epithelial–Mesenchymal Transition; a process in which epithelial cells acquire invasive, mesenchymal traits.
**DEG**	Differentially Expressed Gene; shows statistically significant gene expression differences.
**GO**	Gene Ontology; a standardized framework for classifying gene functions.
**GSEA**	Gene Set Enrichment Analysis; identifies functional gene sets enriched under specific conditions.
**scFEA**	Algorithm to infer core metabolic fluxes at the single-cell level.
**Slingshot**	Trajectory inference tool for pseudotime analysis of cell differentiation.
**dyno**	R package for reconstructing developmental trajectories from scRNA-seq data.
**PAGA-TREE**	Method for constructing pseudo-evolutionary trees in single-cell data.
**SComatic**	Tool for detecting somatic mutations from scRNA-seq BAM files.

**TCGA**	The Cancer Genome Atlas; a comprehensive public resource for cancer genomic data.
**maftools**	R package to annotate, visualize, and analyze mutation data in MAF format.
**Pseudo-bulk analysis**	Aggregates single-cell data by group to perform bulk-like differential expression analysis.
